# All-Optical Trapping and Programmable Transport of Gold Nanorods with Simultaneous Orientation and Spinning Control

**DOI:** 10.1021/acsnano.4c10264

**Published:** 2024-09-25

**Authors:** José A. Rodrigo, Tatiana Alieva, Vanesa Manzaneda-González, Andrés Guerrero-Martínez

**Affiliations:** † Universidad Complutense de Madrid, Facultad de Ciencias Físicas, Ciudad Universitaria s/n, Madrid 28040, Spain; ‡ Departamento de Química Física, Universidad Complutense de Madrid, Avenida Complutense s/n, Madrid 28040, Spain

**Keywords:** laser trap-and-transport, optical tweezers, gold nanorods, plasmonics, nanomotors

## Abstract

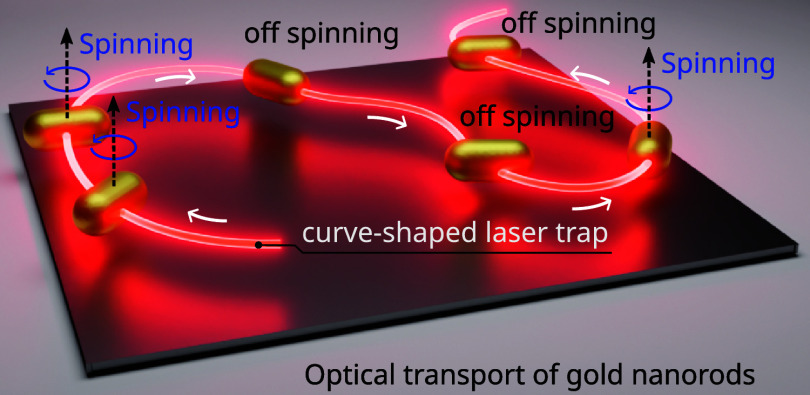

Gold nanorods (GNRs) are of special interest in nanotechnology and biomedical applications due to their biocompatibility, anisotropic shape, enhanced surface area, and tunable optical properties. The use of GNRs, for example, as sensors and mechanical actuators, relies on the ability to remotely control their orientation as well as their translational and rotational motion, whether individually or in groups. Achieving such particle control by using optical tools is challenging and exceeds the capabilities of conventional laser tweezers. We present a tool that addresses this complex manipulation problem by using a curve-shaped laser trap, enabling the optical capture and programmable transport of single and multiple GNRs along any trajectory. This type of laser trap combines confinement and propulsion optical forces with optical torque to transport the GNRs while simultaneously controlling their rotation (spinning) and orientation. The proposed system facilitates the light-driven control of GNRs and the quantitative characterization of their motion dynamics including transport speed, spinning frequency, orientation, and confinement strength. We experimentally demonstrate that remote control of the GNRs can be achieved both near a substrate surface (2D trapping) and deep within the sample (3D all-optical trapping). The motion dynamics of two sets of off-resonant GNRs, possessing similar aspect ratios but different resonance wavelengths, are analyzed to highlight the role played by their optical and mechanical properties in the optical manipulation process. The experimental results are supported by a theoretical model describing the observed motion dynamics of the GNRs. This optical manipulation tool can significantly facilitate applications of light-driven nanorods.

Optical manipulation of nanoparticles facilitates the study of nanoscale mechanical, thermal, and optical effects. For instance, it is frequently applied in scientific fields such as photonics and nanotechnology. Since the invention of the optical (laser) tweezers^1,2^ (in the form of point-like laser traps), optical manipulation has made significant progress in optical confinement, binding, sorting, and transport of particles by utilizing optical forces.^3−6^ Currently, there is intensive research to govern different types of optical forces to dictate the motion dynamics and interactions of nanoparticles immersed in an aqueous solution (e.g., water).^3−6^

Metallic nanoparticles can exhibit strong optical response, enhancing their interaction with light. Thus, they can act as susceptible sensors due to their response to the electromagnetic field being affected by external factors.^4−7^ Their small size, combined with their plasmonic behavior and photothermal properties, enables them to function as mechanical actuators as well.^8−10^ Gold nanoparticles are particularly interesting due to their biocompatibility and stability against oxidation, making them efficient sensors, drug carriers, and photothermal sources in various applications.^8,11^ Moreover, they can be synthesized in different shapes and sizes, which in turn allows for tuning and further exploiting their optical properties. Numerous studies have considered the optical trapping and transport of gold nanospheres;^3,6,12,13^ meanwhile, the optical manipulation of asymmetric nanoparticles such as gold nanorods (GNRs) has been less investigated.^14−18^

GNRs are of high interest for several nanotechnology and biomedical applications^19−22^ due to their anisotropic shape, enhanced surface area, and tunable optical properties in the near-infrared (NIR) region of the light spectrum. In particular, GNRs with optical properties in the NIR region are crucial because NIR light penetrates deeper into biological tissues, enhancing imaging and therapeutic applications like photothermal therapy and drug delivery.^11^ It has been shown that a single off-resonant GNR can be optically trapped in three dimensions (3D) by using the point-like laser trap thanks to intensity-gradient forces arising from the strong focusing of a linearly polarized laser beam,^14^ which does not induce GNR spinning. Note that the 3D optical trapping of a near-resonant GNR is not possible by using a single focused laser beam because its intensity-gradient force is too weak to compensate for the radiation pressure. The use of two counter-propagating focused laser beams can cancel the radiation pressure acting on the illuminated particle, thus enabling 3D trapping of a resonant GNR. For instance, it has been demonstrated that counter-propagating laser beams (generated through focus splitting in a uniaxial birefringent crystal and reflection from a mirror) allow 3D trapping of a resonant GNR with the ability to set it into fast rotation.^23^ This approach is easier to realize than the traditional implementation of a counter-propagating optical trap; however, it requires an especially designed sample chamber comprising a uniaxial birefringent crystal and an appropriate gold-coated slide to act as a mirror.^23^ It has been experimentally demonstrated that near-resonant GNRs can be set into fast rotatory motion (e.g., spinning frequency up to ≈40 kHz in water) in 2D against a substrate, by using circularly polarized single-beam laser focused on it.^16,19^ This type of fast rotatory nanomotor (driven by resonant light) is also of high interest for future nanomechanical as well as biomedical applications involving nanorobotics and nano/microfluidic flow control.^16,17^ More recently, it has been shown that a circularly polarized Laguerre–Gaussian vortex beam spins a GNR (again in 2D, against a substrate) while revolving it around the beam’s symmetry axis.^18^ This combined motion of the GNR makes it a potential nanoscale probe to gain relevant information about the hydrodynamic and thermodynamic properties of its surrounding medium.^18^ When the laser wavelength is close to the longitudinal localized surface plasmon resonance (LSPR) wavelength of GNRs, the increased photothermal heating can significantly alter their shape and the temperature of the surrounding medium. This, in turn, changes the dynamics of the GNRs’ rotational motion. Consequently, optical manipulation of resonant GNRs can be affected by photothermal side effects. However, it has been shown that at low laser power, photothermal heating does not significantly impact the durability and stability of GNR motors.^16,19^

Light-driven rotatory GNRs are particularly interesting in various research fields due to their optical and mechanical properties. Their ability to rotate under the influence of light (through optical torque) makes them valuable for studying nanoscale dynamics and interactions. For instance, this rotational behavior is promising for applications in nanomedicine, where GNRs can be used for targeted drug delivery where their rotation can help in precisely targeting and treating diseased cells.^8,11^ Additionally, rotatory GNRs are promising for sensing due to their sensitivity to changes in the surrounding environment, enabling the detection of minute changes in biological systems. Optical manipulation tools able to control the orientation and rotary motion of GNRs can allow the study of their behavior under various conditions, providing insights into fundamental physical and chemical processes at the nanoscale.

In practice, capturing and controlling the motion of single as well as multiple GNRs in a programmable manner, as needed for specific applications, requires the ability to govern their translational and rotational motion along with their orientation. To achieve this, simultaneous control of different types of optical forces and the optical torque exerted on each GNR is required. This is a challenging optical manipulation problem that has not been previously addressed. In this work, we demonstrate that indeed this is possible by harnessing two types of optical forces available on the curve-shaped laser trap^13,24^ and tuning the optical torque via the light polarization control. Note that this laser trap is completely different from the conventional optical tweezers in the form of a focused laser spot. To create a curve-shaped laser trap, the laser beam has to be focused in the form of a curve, with the ability to independently prescribe the beam’s intensity and phase along it, regardless of the curve’s shape. This laser trap enables programmable particle transport along any trajectory (open and closed curves of arbitrary shape) thanks to the ability to prescribe along it the optical forces responsible for the particle confinement and propulsion.^13,24^ Specifically, the optical confinement arises from the intensity-gradient force acting in the perpendicular direction to the curve, enabling 3D particle trapping. Simultaneously, the prescribed phase-gradient force acts in the tangent direction to the curve for propelling them.^13,24^ Note that in contrast to the intensity-gradient forces, which are conservative forces that drag particles toward the minima of potential energy, nonconservative optical forces such as the phase-gradient one^25^ used here are much versatile being able to pull and move particles.^26^ The term “nonconservative” refers to the fact that part of the energy provided by the light beam is dissipated (absorbed or scattered) to achieve mechanical action.^26^ For the creation of such a complex laser trap with intensity, phase, and polarization prescribed along arbitrary 2D and 3D curves, holographic techniques are used.^13,27,28^ These curve-shaped laser traps offer promising capabilities for programmable transport routing of particles in a robotic fashion, avoiding obstacles and controlling the trajectory of their movement as demonstrated in refs (24, 29, and 30). Note that phase-gradient forces facilitate the optical transport of single and multiple particles making their optical manipulation more versatile. For instance, it has been recently demonstrated that these optical forces can be exerted over metal nanoparticles to facilitate their electrodynamic interaction.^13,31−33^

In this study, we consider curve-shaped laser traps enabling trapping (both in 3D and 2D) and transport of off-resonant GNRs along any trajectory with simultaneous control of their rotatory motion and orientation. Specifically, we use curve-shaped laser traps (in the NIR region, laser wavelength at 1064 nm) with distinct geometries, in the form of ring and square circuits, as a test benchmark ensuring generality. We also utilize a point-like laser trap to demonstrate that 3D trapping of a spinning off-resonant GNR is also possible. The control of the laser polarization allowed us to halt the spinning of the transported GNRs and align them according to the direction of linear polarization of the laser trap. This control over the translational and rotational motions is achieved for off-resonant GNRs not too far from their LSPR. It can also be achieved in the case of near-resonant GNRs, but only against a substrate (2D trapping). The proposed setup allows the generation of these distinct laser traps, controlling the laser polarization in real time, detecting the position and orientation of the GNR as well as measuring its spinning frequency. A theoretical model and numerical simulations are also presented, supporting the experimental results.

## Results and Discussion

### Optical Forces and Torques Exerted over Nanorods

Here, we consider optical forces and torques acting over a particle in the electric dipole approximation,^34^ which is valid for a nanoparticle smaller than the wavelength of the illumination wave (λ = 1064 nm in our case).

The time-averaged optical force exerted by a monochromatic wave field over the nanoparticle is given by^35,36^
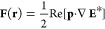
1while the exerted time-averaged spin torque is expressed as^35,36^
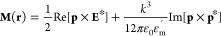
2where **r** = (*x*, *y*, *z*) is the position vector, the symbol * stands for complex conjugate, **p** = α̂**E** is the electric dipole moment with α̂ being the particle’s polarizability tensor, **E** = **E**_0_(**r**) exp(iφ(**r**)) = (*E*_*x*_, *E*_*y*_) is the electric field distribution, and  is the wavenumber in the nonabsorbing medium of electric permittivity ε_m_. Note that the international system of units is used. We consider a wave field with a uniform polarization distribution; thus, **E**_0_/|**E**_0_| = cos θ**u**_*x*_ + exp(*i*δ) sin θ**u**_*y*_ describes linear polarized light for δ = *n*π (with *n* being an integer number) and circular for θ = π/4 with δ = ± π/2 (elliptical polarization otherwise).

The polarizability of a GNR is anisotropic, and it can be written as
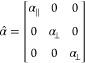
3in Cartesian coordinates associated with the principal axes of the polarizability tensor. The longitudinal component (α_∥_) and transverse one (α_⊥_) are complex-valued functions: α_∥,⊥_ = α_∥,⊥_*′*(λ) + *i*α*″*_∥,⊥_(λ), that depend on the particle (its shape and electric permittivity ε_GNR_) and its surrounding medium’s permittivity (ε_m_). Here, we have used analytical expressions of α_∥,⊥_ reported in ref (37). Moreover, we suppose that the symmetry axis of the nanorod (e.g., the *x* axis) and the electric field vector (its transverse components) lie in the *xy* plane. Thus, the dipole momentum for the nanorod is given by
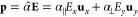
4where **u**_*x*,*y*,*z*_ are the unit vectors in the direction of the axes *x*, *y*, and *z*.

The optical torque for the case of linearly polarized (LP) light, **E** = *E*(cos θ, sin θ), is expressed as
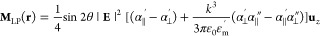
5that tends to align the nanorod along the polarization direction of the electric field or perpendicular to it (i.e., θ = *n*π/2). Indeed, discarding the recoil part of the optical torque (see the second term, which is proportional to *k*^3^), the nanorod is oriented parallel to the direction of the electric field polarization if α_∥_*′* > α_⊥_*′* and perpendicular to it if α_∥_*′* < α_⊥_*′*.^38^ For circularly polarized (CP) light, the optical torque is given by
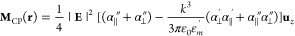
6In practice, it is useful considering the normalized optical torque, **M**_n_(**r**) = **M**(**r**)/*I*, where the irradiance of the wave illuminating the nanorod can be expressed as .

The optical force exerted over a nanoparticle (see eq 1) splits into two terms: one proportional to the intensity gradient (∇|**E**|^2^) of the focused light beam and the other one proportional to its phase gradient (∇φ), which are responsible for the optical confinement and propulsion of the particle, respectively.^13^ Specifically, in the case of LP light, the optical force exerted over the GNR is given by
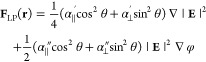
7The first term in eq 7, further referred to as **F**_∇I,LP_, corresponds to the intensity-gradient force that can be attractive or repulsive depending on the sign of the real part of α_∥,⊥_(λ). In our case, α*′*_∥,⊥_(λ) > 0, and the nanorod can be optically trapped by the attractive intensity-gradient force. The second term in eq 7 is the optical propulsion force **F**_∇φ, LP_, which is proportional to the optical current |**E**| ^2^∇φ of the beam. The propulsion force can drive the optical transport of any particle along a curved trajectory **r**_c_ in 2D and 3D as reported in ref (13). Since α*″*_∥,⊥_(λ) is always a positive value, the sign of the propulsion force is only given by the sign of the phase gradient ∇φ(**r**_c_) prescribed along the transport trajectory **r**_c_. For instance, in the case of a transport circuit in the form of a ring of radius *R*, parametrized as **r**_c_ = *R*(cos Θ, sin Θ), the phase gradient is given by ∇φ(**r**_c_) = (*Q*/*R*)**u**_Θ_, where **u**_Θ_ = (−sin Θ, cos Θ) is the unit vector tangent to the ring and *Q* is an integer number whose sign defines clockwise (*Q* < 0) or counterclockwise (*Q* > 0) particle transport.^13^ Interested readers can refer to refs (13 and 24) (see also Methods) for further details about how to generate any curve-shaped laser trap, whose intensity and phase distributions are independently prescribed along the transport trajectory to control the propulsion force on demand. Note that for a stiff optical propulsion force, the speed **v** of the particle is proportional to the applied phase gradient^13,25^ as **v**(**r**) = **F**_∇φ_/γ_t_ ∝∇φ(**r**_c_)/γ_t_, where γ_t_ is the translational drag friction coefficient of the particle^39^ (see Methods).

In the case of CP light, the confinement and propulsion forces are proportional to real and imaginary parts of the particle’s polarizability as follows:
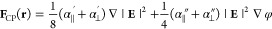
8Let us emphasize that the real and imaginary parts of the polarizability α_⊥_ are much smaller than the ones corresponding to α_∥_ at the considered laser wavelength. Thus, α_⊥_ can be ignored in the estimation of the optical force and torque strengths. By comparing the optical forces given by eqs 7 and 8, it is easy to determine the strength ratio for the driving force as |**F**_∇φ,LP_|/|**F**_∇φ,CP_| = 2, predicting an enhanced optical propulsion of the particle for LP curve-shaped laser traps with respect to the CP one. In other words, this result predicts that the speed of the transported particle is twice as high for linear polarization than for the case of circular polarization at the same laser power. Analogously, the strength ratio for the optical confinement force fulfills |**F**_∇*I*,LP_|/|**F**_∇*I*,CP_| = 2, which indicates that the confinement force is twice as high for linear polarization than for circular polarization for the same laser power.

### Sets of GNRs and Sample Preparation

Here, two sets of cylindrical GNRs of different volumes (but similar aspect ratios), both dispersed in deionized water, have been considered. The first one, referred to as GNR_1_, has been purchased from Nanopartz (A12-70-808-CTAB-DIH-1-1): CTAB-capped GNRs whose average (representative) length is *L* ≈ 162 nm and diameter is *D* ≈ 65 nm (aspect ratio of 2.5, longitudinal LSPR peak at ≈850 nm, see Figure S1). For this representative GNR, the calculated polarizability (according to ref (37)) at our laser wavelength is α_∥_*′*(λ) = 1.45 × 10^–31^ Cm^2^/V and α*″*_∥_(λ) = 0.52 × 10^–31^ Cm^2^/V. The corresponding normalized optical torque is *M*_n,CP_(λ) = 7.1 pN nm/mW μm^–2^ for circular polarization (see Figure S1). The second set, GNR_2_, of CTAB-capped GNRs has been fabricated by us using a seed-mediated anisotropic growth method^40^ (see the Supporting Information). The representative nanorod’s length and diameter of the GNR_2_ set are *L* ≈ 90 nm and *D* ≈ 33 nm (aspect ratio of 2.7, longitudinal LSPR peak at ≈740 nm, see Figure S2). In this case, the calculated values of polarizability are significantly lower, α_∥_*′*(λ) = 0.18 × 10^–31^ Cm^2^/V and α*″*_∥_(λ) = 0.01 × 10^–31^ Cm^2^/V, due to the blue-shifted longitudinal LSPR peak centered at λ_LSPR_ = 740 nm. Consequently, the calculated normalized optical torque is about 36× lower for GNR_2_ than for GNR_1_ at our laser wavelength. Let us underline that the considered laser wavelength is far enough from the longitudinal LSPR peak of the GNRs: Δλ_LSPR_ = 214 nm and Δλ_LSPR_ = 324 nm for GNR_1_ and GNR_2_, respectively. The significant difference in the values of electric polarizability and, therefore, in the optical forces and torques of these off-resonant GNR sets yields different motion dynamics studied in the following sections. Figures S1 and S2 display the polarizability and normalized optical torque as a function of the illumination wavelength, for both sets GNR_1_ and GNR_2_. The sample, a droplet of diluted aqueous GNR suspension (≈ 2 μL), was placed between a glass microscope slide and a coverslip separated by ≈50 μm spacer tape.

### Optical Manipulation and Detection Systems

**Figure 1:**
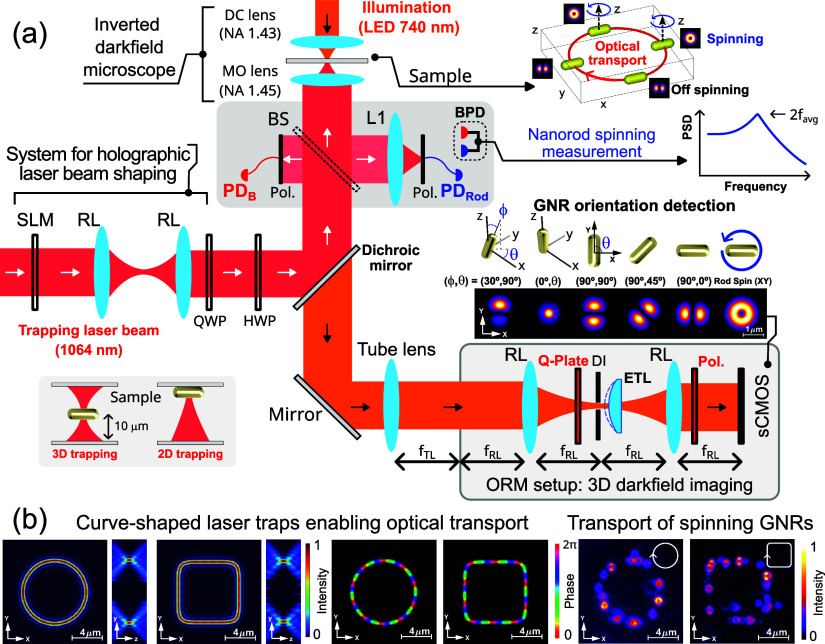
(a) Sketch of the optical setup required for the optical manipulation of the GNRs, their orientation detection, and measurement of their spinning frequency (by using a fiber-coupled balanced photodetector device, BPD). The spatial light modulator (SLM) allows the holographic generation of the laser traps. The optical refocusing module (ORM) allows for 3D darkfield imaging (through optical axial scanning), along with 3D orientation detection of the GNRs (thanks to a Q-plate, as indicated in the ORM inset). (b) Intensity and phase distributions of the considered curve-shaped laser traps (ring and square circuits) are shown along with the measured darkfield images of multiple GNRs transported along them. See Methods for further details about acronyms and setup characteristics.

The multifunctional experimental setup used for the optical trapping and transport of GNRs along the target circuit comprises an infrared laser (λ = 1064 nm in vacuum) and a holographic beam shaping system attached to an inverted darkfield microscope, as sketched in Figure 1a. This holographic system enables the generation of the curve-shaped laser trap as well as the point-like one. Specifically, the trapping laser beam is created by modulating an input collimated laser beam with a phase-only computer-generated hologram addressed onto a programmable spatial light modulator (SLM, see Methods) as reported in ref (13). This modulated laser beam is redirected toward the inverted microscope, whose objective lens (MO, Nikon CFI 100×, 1.45 NA) focused it in the form of a curve-shaped laser trap (or a point-like trap if needed). To demonstrate the optical transport of GNRs, we have considered ring- and square-shaped laser traps, whose intensity and phase distributions prescribed along them are shown in Figure 1b. The ring trap has a radius of *R* = 4 μm and a charge of *Q* = −10 (i.e., a number of ten 2π phase shifts along the curve). The side of the square trap has a length of 2*R* = 8 μm and a charge of *Q* = −10 as well. The dichroic mirror (Thorlabs DMSP950) redirects the laser beam to the sample, and it also prevents the camera (sCMOS, Hamamatsu, Orca Flash 4.0, operating at 1 kHz) from back-reflection laser saturation. The linear polarization of the trapping laser beam can easily be converted to circular polarization by using a tandem of quarter- and half-wave plates (QWP and HWP, see Methods) placed before the dichroic mirror, as depicted in Figure 1a. A proper rotation of the QWP and HWP allows compensating the ellipticity arising from the polarization-dependent response of the dichroic mirror.^41^ Specifically, by using the QWP-HWP tandem, the measured phase shift δ between the s and p polarization components of the trapping beam (reflected by the dichroic mirror) is δ = 88.7°, which is a value close enough to the ideal one 90° for CP light.

The experimental setup is also equipped with an optical refocusing module (ORM) placed at the exit plane of the microscope as indicated in Figure 1a, which has been developed by us in previous work.^13^ This ORM system enables 3D darkfield imaging of the sample, and it is used here for precise optical (nonmechanical) focusing of the image plane. The optical axial focusing is achieved by using an electrically tunable lens (ETL, varifocal lens Optotune EL-10-30-C) placed as indicated in Figure 1a. Under darkfield illumination, unscattered light from the sample is eliminated. In our case, we employed darkfield illumination with a wavelength of 740 nm (near the longitudinal LSPR of the GNRs), focusing it on the sample using a 1.43 NA darkfield condenser (Nikon). Let us underline that the darkfield iris (DI) was mounted alongside the ETL (in the ORM, see Figure 1a) rather than the focusing MO lens to prevent filtering of the laser trapping beam projected onto the entrance back aperture of the MO lens.

To detect the position and 3D orientation of the GNRs, we have incorporated a Q-plate (Thorlabs, vortex retarder WPV10L-705) and a polarizer in the ORM (see Figure 1a and Methods). As experimentally proved in ref (42), the use of the Q-plate and polarizer allows for single-shot 3D orientation imaging (in real time) of nanorods under Brownian motion. We have adopted the technique reported in ref (42) to analyze the 3D orientation of the GNRs optically transported by our laser traps. The combined use of the ORM and the Q-plate systems enables straightforward scanning of the sample at different depths to analyze the 3D orientation of the GNRs. The expected darkfield images of GNRs with and without spinning are shown in Figure 1a (see the ORM inset).

To analyze the rotatory dynamics of a single GNR in the laser trap, we used a polarization-sensitive setup placed before the MO lens as indicated in Figure 1a. It allows measuring the time-series intensity fluctuations of the laser backscattered by the GNR, which is collected by a polarizer using a fiber-coupled gain-balanced amplified photodetector device (BPD, Thorlabs PDB450C-AC, operating up to 1 MHz). The BPD consists of two fiber-coupled photodetectors (PD_B_ and PD_Rod_ in Figure 1a) working as a balanced receiver by subtracting the two input signals from each other, resulting in the cancellation of the common mode noise. This type of noise cancellation is crucial for detecting small differences in optical power between the two optical input signals. Therefore, the BPD allows suppressing the common intensity fluctuations caused for example by both the SLM and laser devices from the intensity fluctuations caused by the rotatory dynamics of the GNR. Specifically, the intensity of the light backscattered by the GNR is collected by the fiber-coupled photodetector (input PD_Rod_ of the BPD device) as a time-series intensity described by^17,19^
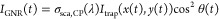
9where σ_sca,CP_(λ) is the GNR scattering cross-section (for circular polarization at the laser wavelength), while θ(*t*) is the rotation angle of the GNR (in the focal *xy* plane) relative to the polarizer. The nanorod angle variations θ(*t*) are thus converted to fluctuations in *I*_GNR_(*t*) due to the nanorods’ highly polarized light scattering. Note that *I*_trap_(*x*(*t*), *y*(*t*)) is the value of the laser trap intensity (irradiance at the focal plane) evaluated at the GNR position (*x*(*t*), *y*(*t*)) for a time *t*. For instance, in the case of a point-like laser trap, its irradiance distribution can be approximated by *I*_trap_(*x*, *y*, *t*) ∝ *I*_peak_(*t*) exp[−2(*x*^2^ + *y*^2^)/*w*^2^] (a Gaussian-like laser spot),^4,43^ where *w* is the beam waist size and *I*_peak_(*t*) ∝ *I*_B_(*t*) with *I*_B_(*t*) being the beam intensity measured at the entrance aperture of the focusing MO lens (see Figure 1a). Temporal fluctuations in beam intensity *I*_B_(*t*) (and therefore in *I*_peak_(*t*)) are caused by the SLM and laser device operation. The signal *I*_B_(*t*) is collected by the second fiber-coupled photodetector (PD_B_), as sketched in Figure 1a. The output signal of the BPD device is *I*_out_(*t*) ∝ *I*_GNR_(*t*) – β*I*_B_(*t*), where β is an intensity-ratio parameter enabling autobalanced operation. Therefore, the balanced output signal *I*_out_(*t*) provides a measure of the time-series intensity fluctuations of the laser backscattered by the GNR free of any fluctuation carried by *I*_B_(*t*). It allows us to obtain a reliable measurement of the time-averaged spinning frequency *f*_avg_ = ⟨dθ/d*t*⟩/2π of the GNR from the power spectral density (PSD) and autocorrelation function (ACF) of the signal *I*_out_(*t*) as discussed in the next section.

### Rotatory Dynamics of GNRs Optically Trapped in 2D and 3D

The rotatory dynamics of near-resonant GNRs have been previously analyzed by using a Gaussian laser beam in the form of a point-like spot^17,19^ as well as in the form of a tiny Laguerre–Gaussian vortex beam with a radius of 0.8 μm,^18^ both focused against a glass coverslip by using a low-numerical-aperture MO lens (NA < 1, dry). Note that in this weak focusing configuration, the axial intensity-gradient force of the laser beam is too weak to compensate for radiation pressure. As a result, only 2D trapping of the GNR can be achieved against the glass coverslip thanks to its Coulomb repulsion. In the absence of Coulomb repulsion, the GNR becomes stuck to the glass coverslip. These types of 2D laser traps have been created (without using an SLM) from a Gaussian laser beam whose wavelength is located at Δλ_LSPR_ ≈ 80 nm from the longitudinal LSPR peak (at its red side) of the GNRs considered in refs (17−19). In contrast, here we consider a strongly focused laser beam (a 1.45 NA oil immersion MO focusing lens) whose intensity-gradient force is strong enough to enable 3D trapping of off-resonant GNRs (with Δλ_LSPR_ ≈ 214 nm, at the red side of their longitudinal LSPR peak). This allowed us to experimentally analyze the rotatory dynamics of GNRs optically trapped in 2D but also in 3D, for both the point-like and curve-shaped laser traps (enabling simultaneous transport).

**Figure 2:**
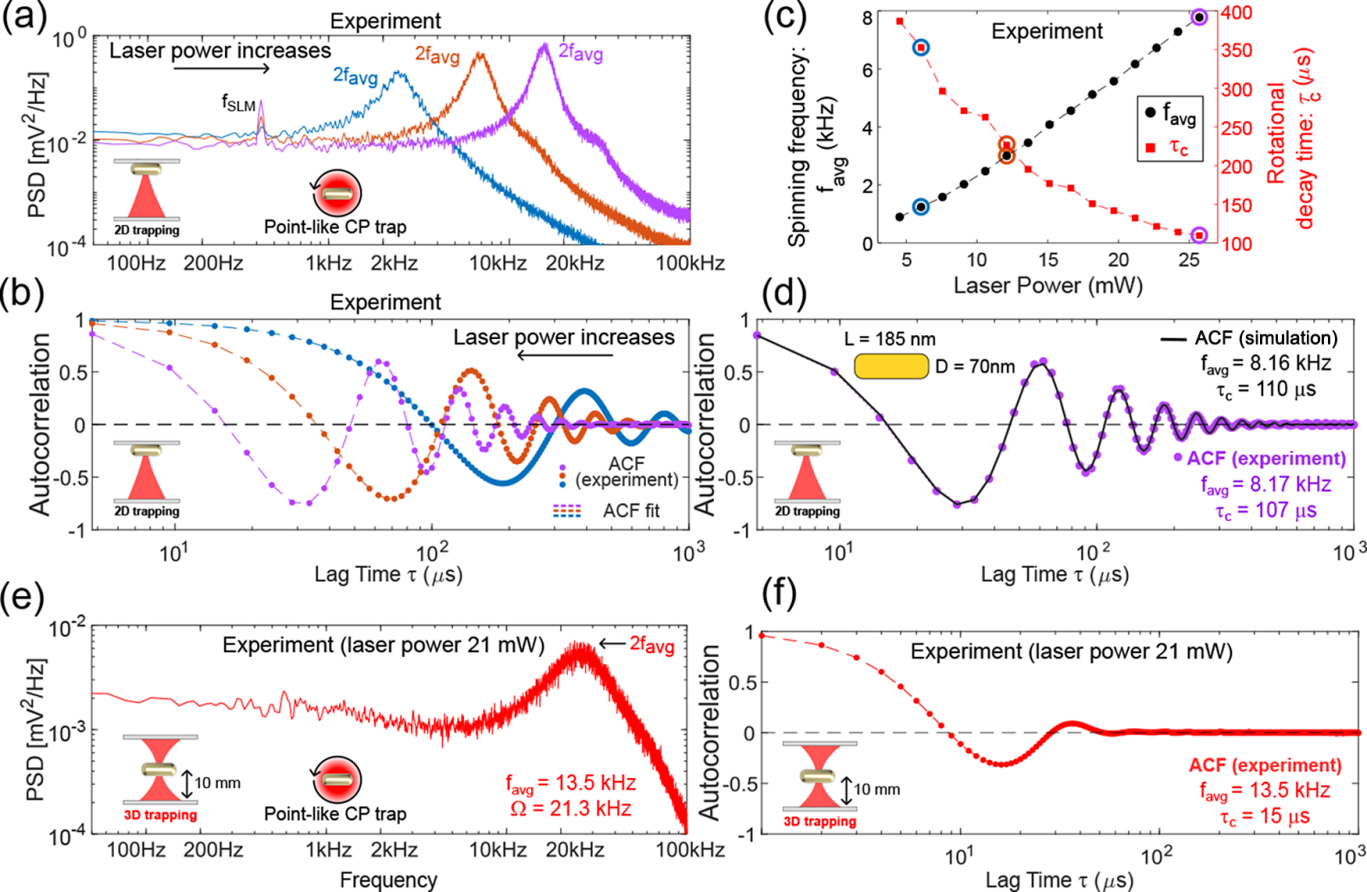
(a) PSDs of the measured intensity fluctuations eq 9 for the same GNR spinning at *f*_avg_ = 1.24, 3, and 7.78 kHz. The small peak observed at a frequency of *f*_SLM_ = 422 Hz corresponds to residual intensity fluctuations caused by the SLM device operation. (b) Fit of the corresponding measured ACFs provides the value of *f*_avg_ and rotational decay time (τ_c_) of the GNR. (c) Measured *f*_avg_ and τ_c_ (estimated from the ACF fitting) are represented as a function of the laser power for the same GNR. Note that in (c), laser power values used in (a) and (b) are also indicated. (d) Measured ACF (scatter plot) of the same spinning GNR (*f*_avg_ = 8.17 kHz) represented along the ACF (black line) obtained from the numerical simulation of the GNR motion. Measured PSD (e) and ACF (f) of a spinning GNR (*f*_avg_ = 13.5 kHz) optically trapped in 3D.

Let us first analyze the rotatory dynamics of GNRs (set GNR_1_) trapped by a point-like laser trap in 2D (against the coverslip). Figure 2a displays the measured PSD for the same GNR for three different values of laser trap irradiance (*I*_peak_). For each value of laser irradiance, the PSD of the measured intensity-time series *I*_out_(*t*) shows a well-defined peak centered at 2*f*_avg_ from which the averaged spinning frequency *f*_avg_ can be estimated. As expected, the value of the averaged spinning frequency increases with the laser power. The fitting of the corresponding experimental ACFs shown in Figure 2b provides a reliable measurement of the averaged spinning frequency *f*_avg_ and a rotational decay time τ_*c*_, which is related to the peak width observed in the PSD. Specifically, the ACF of the measured intensity fluctuations *I*_out_(*t*) can be fitted to
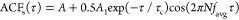
10where *A* is the average intensity and *A*_1_ is the amplitude of the intensity fluctuation, while *N* = 2 stands for cylindrical particles with a 2-fold symmetry as the GNRs.^17,44^ Note that τ_c_ = γ_r_/*N*^2^*k*_B_*T*_r_ is the autocorrelation decay time, where γ_r_ is the rotational friction coefficient of the GNR, while *T*_r_ is the effective rotational Brownian temperature and *k*_B_ is the Boltzmann constant.^17^ The autocorrelation decay time τ_*c*_ provides an estimation of the rotational fluctuation of the GNR, and it relates to the PSD rotational peak width as Ω = (πτ_c_)^−1^ (i.e., the rotational decay rate). The fitting of the experimental ACF, displayed in Figure 2b, provides the following information on the GNR rotatory motion: *f*_avg_ = 1.24 kHz (τ_*c*_ = 353 μs and Ω = 0.9 kHz) for irradiance 8.3 mW/μm^2^, *f*_avg_ = 3 kHz (τ_c_ = 226 μs and Ω = 1.41 kHz) for 16.6 mW/μm^2^, and *f*_avg_ = 7.78 kHz (τ_c_ = 109 μs and Ω = 2.91 kHz) for 34.6 mW/μm^2^. These results demonstrate that the trapped off-resonant GNR exhibits stable rotatory motion with high spinning frequencies. Figure 2c displays the values of the measured averaged spinning frequency *f*_avg_ and rotational decay time τ_c_ as a function of the laser power, measured at the entrance back aperture of the MO focusing lens. A linear increase of the spinning frequency *f*_avg_ is observed, as expected.

The numerical simulation of the translational and rotational motion of a GNR trapped in 2D can provide useful information about the underlying physical mechanisms governing the light-driven behavior of the GNR, as pointed out for example in refs (17, 45, and 46). The translational and rotational motion of a GNR trapped in 2D is often modeled by assuming that both are in-plane motions (see Methods). These motions are developed under the action of the optical force/torque and stochastic fluctuating force/torque characterized by effective Brownian rotational *T*_r_ and translational *T*_t_ temperatures.^17^ The focused point-like laser spot is larger than the GNR, and its isotropic Brownian translational motion exposes the GNR to the spatially varying trap intensity *I*_trap_(*x*, *y*) (i.e., a Gaussian-like laser spot). This is responsible for a spatially varying optical torque *M*_CP_(*x*, *y*) = *M*_n,CP_(λ)*I*_trap_(*x*, *y*), which has also been included in the numerical simulation (see Methods). Thus, this varying optical torque yields rotation frequency fluctuations apart from the rotational Brownian motion effects. The expected averaged spinning frequency is *f*_avg_ = *M*_CP_/2πγ_r_ because the time average of the Brownian torque is zero,^17^ where γ_r_ = γ_r_(*T*_r_) is the rotational drag friction coefficient. In the simulation, we have used analytical expressions for the translational and rotational drag friction coefficients of a nanocylinder.^39^

Our numerical simulation incorporates this temperature-dependent motion model to predict both the translational and the rotational dynamics. The main input parameters of the simulation include the size of the GNR (i.e., the length and diameter of a nanocylinder), the applied laser irradiance and its wavelength, and the properties of the surrounding medium (such as its dynamic viscosity). This enables prediction of the time-series intensity fluctuations (eq 9) caused by the GNR motion, from which the ACF can be computed and compared with the measured one. It allowed us to estimate the expected size of the trapped GNR and its temperature. Indeed, Figure 2d displays the ACF obtained from the simulation (solid black line) that fits well to the measured ACF (scatter plot) for a GNR with a diameter of *D* = 70 nm and a length of *L* = 185 nm, which belongs to the histogram of the measured size of the GNR_1_ set provided in Figure S1. For this GNR, the value of the normalized optical torque is 21 pN nm/mW μm^–2^, which is ≈3× higher than the one corresponding to the representative size of the GNR_1_ set (62 × 165 nm^2^). Specifically, the fitting of the ACF to eq 10 in each case provides the following spinning frequency *f*_avg_(Sim.) = 8.16 kHz (τ_c_ = 110 μs) for the simulation that is in good agreement with *f*_avg_(Exp.) = 8.17 kHz (τ_c_ = 107 μs) for the experiment (for an irradiance of 6 mW/μm^2^). The corresponding estimated values of the effective translational and rotational Brownian temperatures are *T*_t_ ≈ *T*_0_ + 61 K and *T*_r_ ≈ *T*_0_ + 92 K (with *T*_0_ = 298 K being the thermal bath temperature, see Methods), respectively. Note that for the lower value of laser irradiance (2 mW/μm^2^), the same GNR spins at *f*_avg_ = 1.24 kHz and the temperatures are *T*_t_ ≈ *T*_0_ + 20 K and *T*_r_ ≈ *T*_0_ + 30 K. These temperature values indicate that the optical heating of the GNR is sufficiently low (as expected for an off-resonant GNR), which in turns prevents the amplification of the Brownian fluctuations.^17^ The good agreement between the experimental and simulation results validates the considered temperature-dependent motion model to predict the dynamics of the GNRs trapped in the considered 2D laser trap.

Our optical trapping setup allows optically trapping the GNR in 3D far enough from the sample’s walls (see Methods). In Figure 2e,f are displayed the measured PSD and ACF, respectively, of a GNR optically trapped at a depth of 10 μm from the bottom glass coverslip. The fitting of the measured ACF to the expected one in eq 9 gives an average spinning frequency *f*_avg_ = 13.5 kHz and a rotational decay time τ_c_ = 15 μs (a peak width of Ω = 21.2 kHz). To stably trap such small nanoparticles in 3D, it requires higher laser power than in the previous 2D trapping experiments. This increase of the laser power gives a high value of the spinning frequency as expected. The low value of the rotational decay time indicates that the spinning of the GNR is less stable (see also ACF in Figure 2f). This is mainly caused by axial position fluctuations of the GNR in the laser trap that expose it to a spatially varying trap intensity *I*_trap_(*x*, *y*, *z*) (and thus varying the optical torque) of the 3D laser spot. It yields more fluctuations in the rotation frequency of the GNR than in the case of 2D laser trapping, which suppress the axial translation of the GNR. Nevertheless, the stiffness of the optical trap is high enough to strongly confine the GNR for several minutes (>20 min). This representative experimental result, reproducible for other GNRs from the set GNR_1_, proves that simultaneous spinning and 3D trapping of GNRs are indeed possible. In the next section, we experimentally demonstrate that it is also possible to optically transport spinning GNRs by using 2D and 3D laser traps.

The same experimental study was conducted for the second set, GNR_2_. In this case, the observed rotatory motion of the GNRs is unstable, and a well-defined peak in the PSD has not been obtained for the same experimental conditions (see Figure S2). This is explained by the low value of the imaginary part of the polarizability (α_∥_(λ)), which is 52× lower than the corresponding value for the set GNR_1_. Thus, the mechanical effect of the optical torque exerted on GNR_2_ is insufficient to override the Brownian rotational fluctuations.

### Optical Transport of GNRs

**Figure 3:**
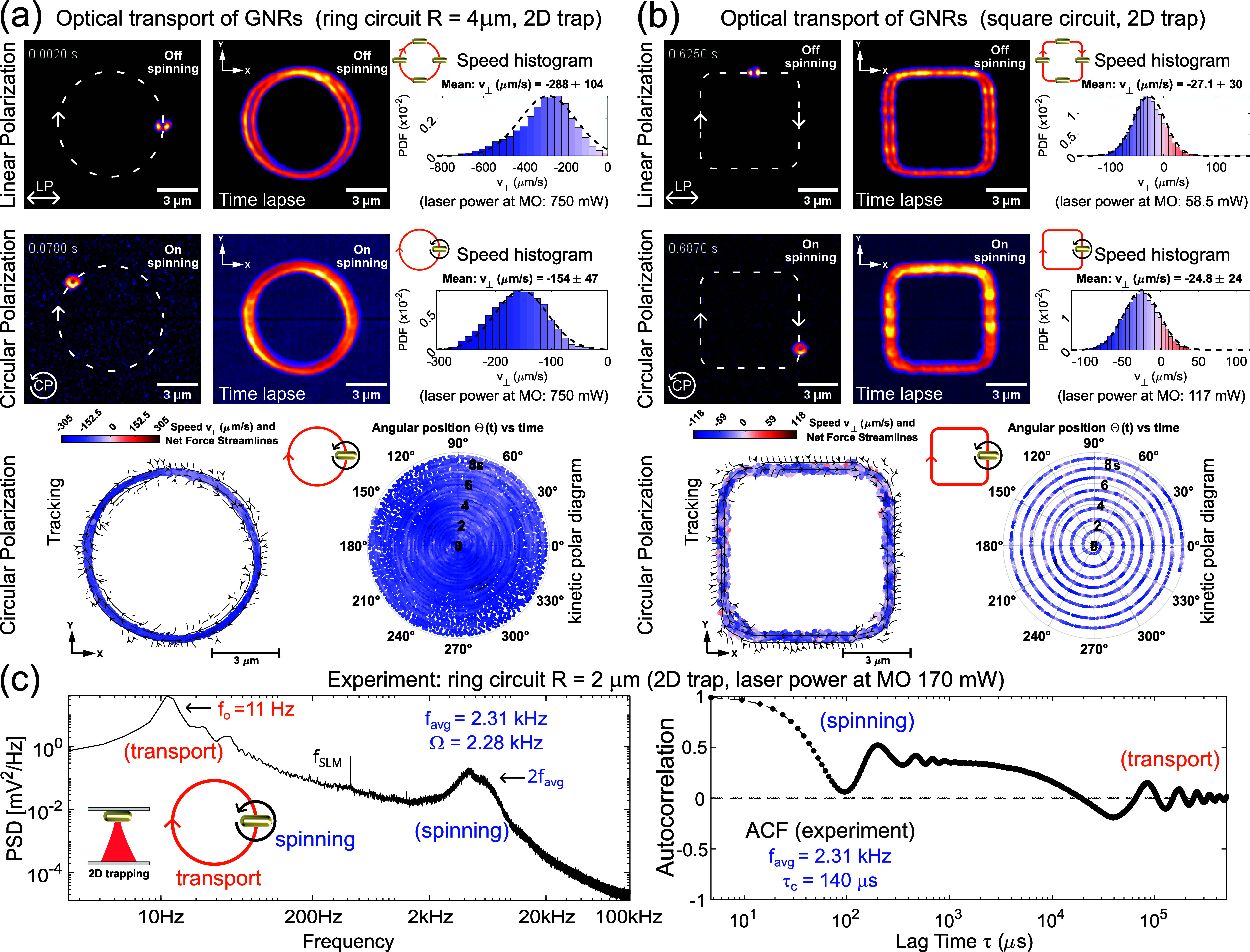
Experimental results. Optical transport of a single GNR (average size 65 × 162 nm^2^) along the ring circuit (a) and square circuit (b) for both linear (LP) and circular (CP) polarization. The value of the mean transport speed (see histograms) is indicated for each case (LP and CP). The tracking data of the GNR as well as the kinetic polar diagram are displayed for CP, for both the ring circuit (a) and square circuit (b). (c) PSD and ACF were measured in the case of a spinning GNR that travels along a ring laser trap with radius *R* = 2 μm and charge *Q* = −4. The fitting of the measured ACF (scatter plot) to the expected one (eq 11) provides the values of the spinning frequency (*f*_avg_ = 2.31 kHz) and orbital frequency (*f*_0_ = 11.7 Hz) in this case.

Let us first demonstrate the optical transport of the GNRs in 2D (set GNR_1_, against the coverslip surface) on the example of a ring circuit and a square circuit displayed in Figure 3a,b, respectively. In the experiment, we have used both LP and CP laser traps in order to illustrate the simultaneous control of the optical torque and propulsion force exerted over the GNRs (see the first and second rows of Figure 3a,b). In the case of the LP laser traps, a restoring torque is exerted aligning the GNR along the corresponding polarization direction, while for the circular polarization, the optical torque (see eq 6) tends to spin the GNR around the direction of incidence (*z* axis). We have used horizontal linear polarization (along the *x* axis) and therefore the GNRs result aligned along it in the whole circuit regardless of its shape, as observed in the displayed darkfield images (see also time lapse images) of Figure 3a,b. For the considered CP laser traps, the spinning frequency of the GNR is higher than the camera frame rate. Therefore, the spinning GNR exhibits a doughnut-like shape in the darkfield image resulting from the time-averaged rotation during the camera exposure time, as observed in the second row of Figure 3a,b for each circuit. The recorded videos of the experiment, for the GNR transported in the ring trap (Figure 3a), are Videos S1 and S2 for LP and CP, respectively. For the square trap (Figure 3b), the corresponding videos are Videos S3 and S4 for LP and CP, respectively.

To obtain more detailed information about the GNR translational motion dictated by the optical propulsion force, the radial **v**_ρ_ = *v*_ρ_**u**_ρ_ and azimuthal (orbital) **v**_⊥_ = *v*_⊥_**u**_Θ_ components of the speed vector (expressed as **v**(*t*) = **v**_ρ_ + **v**_⊥_) have been analyzed, where **v**_⊥_·**v**_ρ_ = 0. The speed vector **v**(*t*) is computed from the position tracking data, which have been obtained from the recorded video of a single GNR transported along both circuits. We have used the tracking algorithm reported in ref (47). The experimental trajectory of the GNR is shown for each curve-shaped laser trap along with its optical force streamlines (see Figure 3a,b, respectively). The corresponding histograms for the azimuthal (orbital) speed *v*_⊥_ (given by its probability density function, PDF) are also displayed in Figure 3a,b. Specifically, the orbital speed reveals a Gaussian distribution centered at *v*_LP_ = −288 μm/s and *v*_CP_ = −154 μm/s (negative sign indicates clockwise motion) in the case of the ring-shaped laser trap with linear and circular polarization, respectively. The obtained speed ratio is *v*_LP_/*v*_CP_ = 1.9, which confirms the theoretical prediction that the speed of the particle is twice as high for linear polarization than for circular polarization for the same laser power. Another significant finding is the achieved high-speed values that provide evidence of the rapid optical transport of GNRs. To illustrate the dependence of the GNR’s speed on the polarization and laser power, we used half the laser power for linear polarization in the square-shaped laser trap (first row in Figure 3b) compared to circular polarization (second row in Figure 2b). Specifically, the speed histogram is centered at *v*_LP_ = −27.1 μm/s and *v*_CP_ = −24.8 μm/s for a laser power of 58.5 and 117 mW, respectively. In other words, to achieve the same transport speed for each case, it was necessary to use half the laser power for linear polarization as expected. The trajectory of the GNR fits to these curve-shaped laser traps, as observed in the third row of Figure 3a,b, where a kinetic polar diagram is displayed for each trap. This kinetic polar diagram represents the angular position of the GNR transported along the circuit as a function of time, where the color of the scatter plot indicates the measured speed *v*_⊥_ of the GNR. These experimental results quantitatively confirm a stable directed GNR translational motion dictated by a stiff driving phase-gradient force prescribed along the transport circuit, regardless of its shape.

The spinning frequency of a GNR transported along a CP ring-shaped laser trap (with radius *R* = 2 μm and *Q*_o_ = −4) can be estimated from the measured ACF, as in the case of the point-like laser traps previously analyzed. Specifically, the measured PSD and ACF data are displayed in Figure 3c. As observed, the PSD exhibits a peak centered at 2*f*_avg_ ≈ 4 kHz (GNR spinning) and another one at ≈11 Hz corresponding to the orbital frequency *f*_0_ (i.e., number of turns per second) of the GNR transported along the ring. The measured ACF displayed in Figure 3c shows two well-separated oscillators corresponding to the spinning and orbital rotation of the GNR. Indeed, the measured ACF function can be fitted using the sum of two damped oscillators describing the orbital transport and spinning of the GNR independently, as follows:
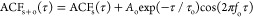
11where the first oscillator corresponds to the ACF_s_(τ) given by eq 10 and the second one has an orbital frequency *f*_0_ and decay correlation time τ_o_. The fitting of the measured ACF provides a spinning frequency *f*_avg_ = 2.31 kHz (with τ_c_ = 140 μs and Ω = 2.28 kHz) and an orbital frequency *f*_0_ = 11.7 Hz (with τ_o_ = 151 μs). The increase in the rotational peak width Ω = 2.28 kHz, obtained from the ACF fitting and well noticed in the measured PSD (see Figure 3c), indicates that the spinning frequency of the transported GNR fluctuates probably due to slight nonuniform intensity in the ring trap.

**Figure 4:**
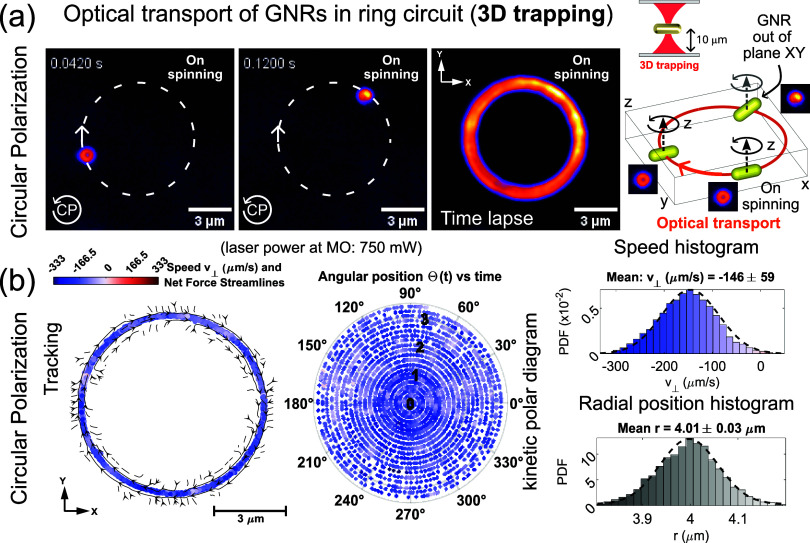
Experimental results. (a) Optical transport of a spinning GNR trapped in 3D (at a trapping depth of ∼10 μm) by a ring laser trap with circular polarization (CP), see also Video S5. (b) Corresponding tracking position data of the GNR, the kinetic polar diagram, and the speed histogram are displayed. The displayed histogram of the measured radial position of the GNR allows estimating the radial trap stiffness: κ_r_ = 3.6 pN/μm. The mean orbital speed of the GNR is *v*_CP_ = −146 μm/s, indicating a stiff propulsion force governing the transport of the spinning GNR.

Here, we also experimentally demonstrate that 3D optical trapping and transport of spinning GNRs is possible (see Figure 4 and Video S5). Specifically, the optical transport of the GNR along the CP ring laser trap is demonstrated at a trapping depth of ∼10 μm (as in the case of the point-laser trap), far away from the chamber walls (e.g., microscope’s coverslip glass), avoiding any interfering substrate effects. As in the previous experiments, the darkfield image of a spinning GNR exhibits a doughnut-like shape resulting from the time-averaged rotation during the camera exposure time. This result indicates that the GNR displays a spinning motion contained in the *xy* plane, as in the case of 2D trapping against the glass coverslip. The time lapse image shown in Figure 4a is also similar to the one obtained for 2D trapping (Figure 3a). This in-plane spinning of the GNR is stable along the whole ring except for a small region in the first quadrant of the ring (located around Θ ≈ 70°), where the GNR displays an out-of-plane rotation as indicated in the 3D inset of Figure 4a. This eventual out-of-plane rotation of the GNR is caused by a diminished 3D optical entrapment in this region of the ring trap. It is known that the residual wavefront aberrations, mostly defocus and astigmatism arising from slight beam misalignment, can degrade the beam focusing on certain regions within a laser trap. As a result, the deteriorated beam focusing diminishes the axial optical trapping of the GNRs. Nevertheless, this slight degradation of the axial confinement does not affect the optical transport of the GNR, which is stable and well-directed along the whole ring trap, as demonstrated by the corresponding tracking data and kinetic polar diagram displayed in Figure 4b. Specifically, the histogram of the measured orbital speed reveals a Gaussian distribution centered at *v*_CP_ = −146 μm/s. This is the first experimental demonstration that a spinning GNR can be simultaneously transported and trapped in 3D. The histogram of the measured radial position of the GNR trapped in the ring trap is also shown in Figure 4b. It exhibits a Gaussian distribution centered at a mean radial position of *r*_avg_ = 4 μm that coincides with the radius *R* of the ring trap. Let us underline that for the ring laser trap, the intensity-gradient force can be expressed by Hooke’s law as *F*_∇*I*_(*r*) = −κ_r_(*r* – *R*) acting in the radial direction *r* (i.e., perpendicular direction to the ring),^13^ with κ_r_ being the radial stiffness (spring constant) of the ring trap. Note that the trap stiffness is proportional to the real part of the particle’s polarizability (α*′*) and the laser power (*P*) as κ_r_ ∝ α*′P*∇*I* (see for example refs (14 and 17)). From the standard deviation (σ(*T*_0_) = 34 nm) of this Gaussian distribution, it is possible to estimate the radial stiffness as κ_r_ = *k*_B_*T*_0_/σ^2^ = 3.6 pN/μm, where *T*_0_ = 298 K is the temperature of the surrounding medium (water). This high value of the radial stiffness indicates a strong optical confinement of the GNR in the *xy* plane (perpendicular to the light propagation direction). This also indicates that an eventual out-of-plane rotation of the GNR is due to a diminished axial trap stiffness κ_*z*_ at some parts of the ring trap.

At this point, it is also interesting to compare the values of radial stiffness obtained in the case of 3D and 2D trapping. In the case of 2D trapping experiment (Figure 3a), the measured radial stiffness of the ring trap is κ_r_ = 0.9 pN/μm (for circular polarization), which is one-fourth of the value obtained in the 3D case for the same laser power. This decrease in trap stiffness is due to the trapping beam being focused against the coverslip glass of the sample at a depth of much greater than 10 μm. As is well-known, the degradation of the trapping beam increases at greater focus depths (in this case ∼50 μm) due to spherical aberration,^48^ which explains this decrease in the measured radial stiffness. Here, it is worth mentioning that different experimental techniques to expand the 3D trapping range of gold nanoparticles with diminished beam degradation have been explored in refs (48 and 49). Interestingly, for the case of the 2D ring trap with linear polarization (Figure 3a), the measured radial stiffness is κ_r_ = 1.68 pN/μm at the same optical power. Thus, the measured radial stiffness is almost twice as high for linear polarization than for circular polarization. This experimental result indicates that the confinement force is twice as high for linear polarization than for circular polarization for the same laser power, as predicted by the previously explained theoretical model.

**Figure 5:**
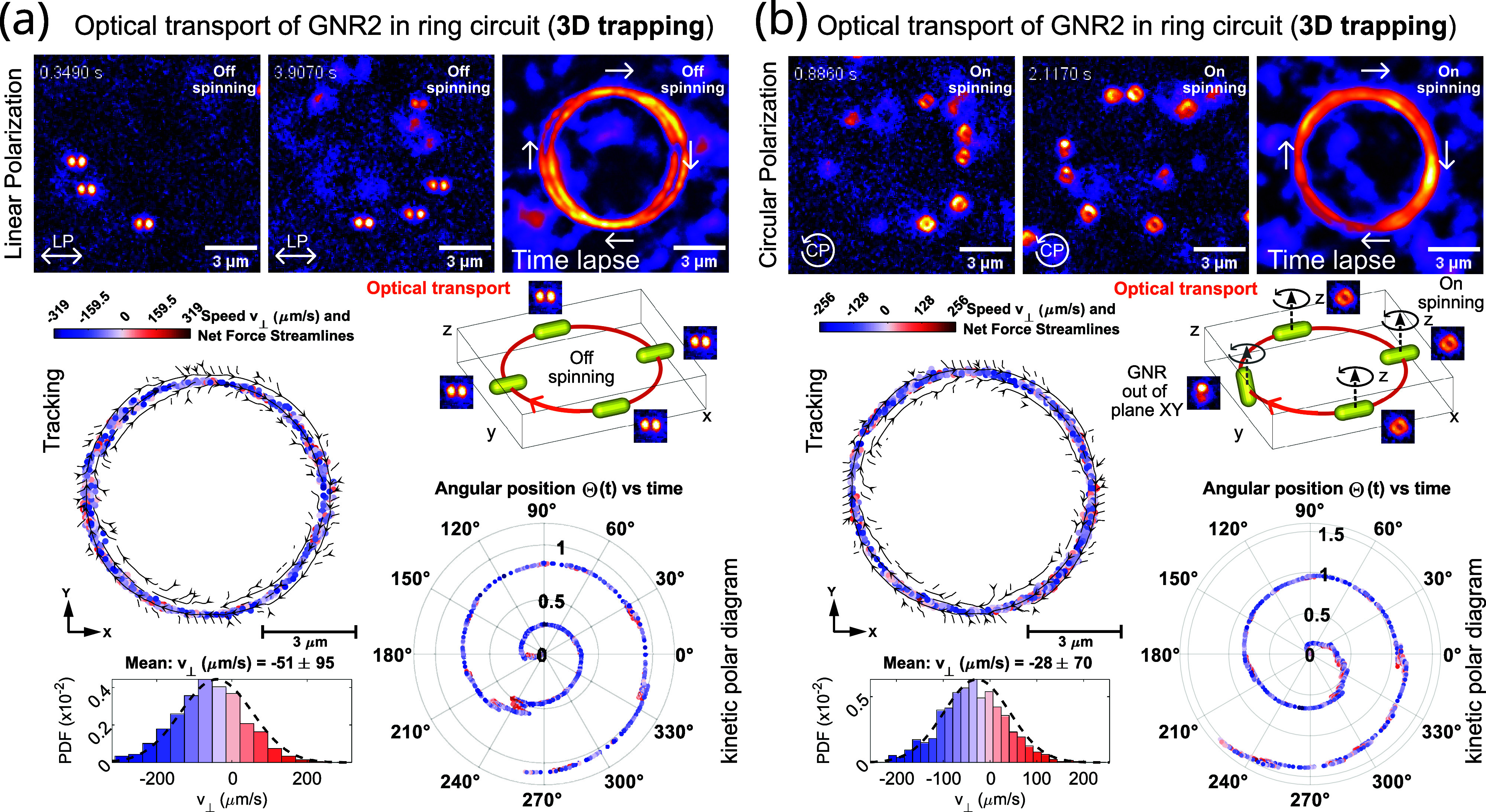
Experimental results. Optical transport of several GNRs (set GNR_2_ with an average size of 33 × 90 nm^2^) along the ring circuit for both LP (a) and CP (b). The tracking data of a single GNR as well as the kinetic polar diagram are displayed in each case, along with the histogram of the measured transport speed. Note that the longitudinal LSPR of GNR_2_ is blue-shifted at λ_LSPR_ = 740 nm. This makes the GNR_2_ less responsive to the optical forces (for the same laser power and wavelength) making its optical transport slower than the previous experiment shown in Figure 4 corresponding to the GNR_1_ set.

To further illustrate the role played by the polarizability of the GNR in the optical transport, let us finally consider the set GNR_2_ (an average size of 33 × 90 nm^2^ and an aspect ratio of 2.7). As previously mentioned, for this set, the nanorod’s polarizability is significantly lower than that for the GNR_1_ set. Thus, the laser power has to be significantly increased to achieve optical trapping and transport as stable as in the previously demonstrated experiments for GNR_1_. In Figure 5, the experimental results obtained for GNR_2_ trapped by the same ring laser trap (and optical power) are displayed. For the LP ring laser trap, the GNR_2_ is aligned to the light polarization direction in the whole transport circuit (Figure 5a), as expected. The GNR_2_ exhibits in-plane directed translational motion with stable rod alignment as observed in the time lapse image and tracking data displayed in Figure 5a. This experimental result shows the ability to optically transport and align the GNR_2_ trapped in 3D, despite its low polarizability α_∥_*′*. However, the optical transport of GNR_2_ along the ring is less stable, as observed in the kinematic polar diagram (see Figure 5a). Moreover, its mean orbital speed *v*_LP_ = −51 μm/s (see the speed histogram) is ≈3× slower than that in the previous experiments (displayed in Figure 4). Let us recall that the propulsion force is proportional to α_∥_^*″*^, which is significantly low for the GNR_2_ set; thus, it explains this slow transport. In the case of the CP ring laser trap, the trapped GNR_2_ is free to rotate driven by the optical torque, as observed in the darkfield images displayed in Figure 4b. The optical transport of the spinning GNR_2_ is less stable and even much slower, at the mean speed of *v*_CP_ = −28 μm/s (see Figure 4b). For the GNR_2_, the obtained speed ratio is *v*_LP_/*v*_CP_ ≈ 1.8, which again confirms that the theoretical prediction about the speed of the nanorod is twice as high for linear polarization than for circular polarization for the same laser power. The diminished optical force and torque exerted over the GNR_2_ (at this laser power) makes its spinning difficult. Nevertheless, from the collected darkfield images, we concluded that the spinning of the GNR_2_ is mostly in the *xy* plane except at some parts of the ring where the GNR_2_ is eventually out-of-plane oriented at an angle of ∼30° with respect to it, which has been indicated in the 3D sketch displayed in Figure 4b to help the visualization. In this experiment, we have also observed several GNR_2_ undergoing a random transition from continuous spinning to discrete rotational jumps. This evidence confirms that the optical torque exerted over the GNR_2_ is much smaller, and thus, it cannot dominate the random (stochastic) nature of the rotational Brownian torque.^17,50^ Indeed, for the GNR_2_ set, the value of the calculated normalized torque is low: *M*_n,CP_(λ) = 0.2 pN nm/mW μm^–2^. The dominance of rotational Brownian diffusion has been further confirmed by fitting the measured ACF (displayed in Figure S2). The frequency width Ω is up to 12× larger than the average spinning frequency. Note that for GNRs with an LSPR wavelength significantly different from the laser wavelength, the resulting optical torque is insufficient to overcome the rotational Brownian diffusion caused by the surrounding medium (e.g., water). Consequently, the rotational motion of such GNRs is unstable compared to those with LSPR wavelengths closer to the laser wavelength. Let us recall that the difference between the wavelengths of the laser beam and LSPR of the GNRs is 214 nm for GNR_1_ and 324 nm for GNR_2_.

## Conclusions

We have experimentally demonstrated 2D and 3D optical trapping and transport of GNRs with the simultaneous control of their orientation and rotatory motion dynamics. To achieve this, we used a curve-shaped laser trap that enables programmable particle transport along any trajectory (transport circuit). This capability arises from the ability to govern both the confinement and propulsion optical forces along the trajectory.^13,24^ The control of the laser trap polarization enables the deactivation or activation of the nanorod’s spinning at a frequency within the kilohertz range (e.g., at rates of 1–20 kHz). The proposed experimental setup comprises several modules that enable the generation of the curve-shaped laser traps (also point-like ones), the detection of the position and orientation^42^ of the nanorod as well as measuring its spinning frequency. The position tracking data of the trapped nanorod enable the estimation of the optical confinement strength (i.e., trap stiffness) and transport speed. This multifunctional experimental setup allowed us to quantitatively study the combined action of the optical torque with the optical confinement and propulsion forces for driving the nanorod’s motion dynamics. The proposed theoretical optical model (for the optical forces and torques), along with a well-known temperature-dependent motion model,^17^ explains the observed translational and rotational dynamics of the transported nanorods.

Let us underline the following important findings and experimental achievements of this work. We have demonstrated that it is possible to set a GNR into rapid spinning while it is optically trapped in 3D by point-like and curve-shaped laser traps. The rotational and translational dynamics of all-optical (3D) and 2D (against the coverslip) trapped GNRs have been compared. We have shown that by using a linearly polarized laser trap, all the transported nanorods are aligned along the light polarization direction (fixed polarization-aligned nanorod orientation), regardless of their speed and the shape of the transport circuit. By using a circularly polarized curve-shaped laser trap, the optical transport of spinning nanorods along the whole circuit has been demonstrated as well. The ability to dictate the orientation and spinning of the nanorod has been analyzed for two sets of off-resonant GNRs with similar aspect ratios but rather different electric polarizability values at the laser trapping wavelength. For both types of nanorods, the experiments show the crucial role played by the nanorod’s polarizability. Specifically, the experimental results confirm the theoretical prediction regarding the nanorod’s transport speed and optical confinement. For the same laser power, the nanorod exhibits twice the speed and confinement when linearly polarized curve-shaped laser traps are used compared to those when circularly polarized ones. For instance, we have demonstrated rapid optical transport of GNRs, at high speeds *v*_LP_ = −288 μm/s and *v*_CP_ = −154 μm/s, for linearly and circularly polarized curve-shaped laser traps, respectively. On the other hand, the experimental analysis of the rotational behavior for each set of GNRs evidences its dependence on both the optical torque and the nanorod rotational drag friction coefficient, which account for optical and dynamic properties, respectively. This fact is particularly relevant for metallic nanorods whose longitudinal LSPR is too far from the laser trap wavelength, resulting in a notably diminished optical torque. We have shown that selecting the appropriate laser wavelength is crucial for achieving stable light-driven translational and rotational motion of GNRs. Prior theoretical analysis of particle absorption, scattering, and optical forces and torques is recommended to ensure programmable control of the GNRs. Comparing the behavior of two types of off-resonant GNRs in the same optical trap highlights the necessity of such analysis (see Figures S1 and S2). Merely increasing laser power cannot offset the low polarizability of particles or the resulting propulsion force and torque. This approach can negatively impact trapping stability and cause photothermal heating, potentially leading to the shape reconfiguration of the particle.

In practice, being able to remotely control the light-driven translational and rotational motion as well as the orientation of a nanoparticle is crucial. The simultaneous characterization of the nanoparticle’s motion dynamics is also required. Such characterization provides valuable insights into the optical forces and torques acting on the particle as well as its interactions with the surrounding medium. We have experimentally proved how to address these challenging problems using the example of GNRs. These types of nanoparticles hold particular relevance as they enable innovative techniques for targeted cellular bioimaging, therapy, and micro/nanorheology.^9,51−53^ We envision that the presented findings will enhance practical applications of light-driven nanorods (made from different materials) as nanoscale sensing probes. Additionally, other types of asymmetric and chiral nanoparticles^54,55^ can also benefit from these advancements.

## Methods

### Experimental Setup

As sketched in Figure 1a, a beam splitter (BS, Thorlabs BSW4R-1064) allows the redirection of the laser backscattered by the GNR for the measurement of time-series intensity fluctuations, required to estimate its spinning frequency. Specifically, the backscattered laser is collected after a polarizer by using a fiber-coupled gain-balanced amplified photodetector device (BPD, Thorlabs PDB450C-AC, operating up to 1 MHz), which is connected to oscilloscope hardware (Liquid Instruments, Moku:Go 30 MHz bandwidth, FPGA computer-connected data logger). Two multimode optical fibers (Thorlabs, M124L02, diameter core of 400 μm and 0.5 NA) redirect the backscattered laser into the BPD inputs. The BS can be removed from the system when there is no need to measure the GNR spinning frequency.

The polarization of the laser beam is controlled by a tandem of a quarter-wave plate (QWP, Thorlabs WPMQ10M-1064) and a half-wave plate (HWP, Thorlabs WPH10ME-1064). For instance, the laser trap polarization automatically switches between left circular polarization (CP) and horizontal linear polarization (LP), and vice versa, by rotating the QWP using a motorized precision rotation mount (Newport URS100BCC).

The darkfield illumination wavelength is 740 nm (LED, CoolLED pE-800). To detect the 3D orientation of the GNRs, we have used a Q-plate (Thorlabs WPV10L-705, vortex plate with charge *m* = 1, designed for a wavelength of 705 nm) and a polarizer (Thorlabs LPVIS050-MP2) incorporated in our imaging system (ORM). The darkfield image encoding the 3D orientation of the GNR (see Figure 1a) has been collected by the camera (sCMOS, Hamamatsu, Orca Flash 4.0, 16-bit gray-level, pixel size of 6.5 μm, operating at 1 kHz). The relay RL and tube lenses have a focal length of 200 mm.

We have used a high-numerical-aperture microscope objective lens (Nikon CFI 100×, 1.45 NA) in combination with an immersion oil with a refractive index of 1.56 (Cargille, refractive index liquids set A). This immersion oil allows us to minimize spherical aberrations and enhance trapping efficiency at the considered trapping plane ∼10 μm above the lower sample’s surface, as reported in refs (13 and 48.)

### Curve-Shaped Laser Trap Generation

A curve-shaped laser trap, in the form of a parametric 3D curve **c**(Θ) = (**r**_c_(Θ), *z*(Θ)) written in cylindrical coordinates (*R*, Θ, *z*) with **r**_c_ = *R*(cos Θ, sin Θ), can be generated by focusing the polymorphic beam^13,27^ given by
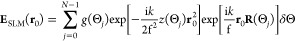
12where **r**_0_ = (*x*, *y*) are spatial coordinates in the objective’s back focal plane. The function *g*(Θ) = |*g*(Θ)| exp[iφ(Θ)] is a complex-valued weight that allows prescribing the beam’s intensity and phase distributions along the curve.^13,27^ The normalization constant f corresponds to the focal distance of the objective lens used to create a laser curve **c**(Θ) around its rear focal plane. Note that Θ_*j*_ = *j*δΘ is used, with δΘ = Θ_max_/*N* being the angle step and *N* being the number of points comprising the discrete representation of the curve **c**(Θ). The parameter Θ_max_ stands for the maximum value of the azimuth angle Θ describing the considered curve. For instance, *N* = 200 points are sufficient to create the curve-shaped laser traps considered in this work (with Θ_max_ = 2π and *z*(Θ) = 0). Let us underline that a curve-shaped laser trap following any nonparametric curve can easily be created as reported in ref (24). In our case, the polymorphic beam eq 12 has been encoded onto the SLM as a phase-only hologram by using a well-known beam shaping technique.^56^

### Numerical Simulations

The translational and rotational motion of a GNR trapped in 2D is often modeled assuming that both are in-plane motions.^17^ Let us recall that we considered a temperature-dependent motion model to predict both the translational and rotational dynamics as reported in ref (17). A relevant aspect of this model is the effective Brownian temperatures *T*_t_ and *T*_r_ associated with the translational and rotational dynamics, respectively. Their values are often assumed as *T*_0_ ≤ *T*_t_ ≤ *T*_r_ ≤ *T*_surf_, where *T*_0_ is the temperature of the thermal bath (e.g., water) and *T*_surf_ = *T*_0_ + Δ*T*_surf_ is the surface temperature of the GNR.^17^ Specifically, *T*_t,r_ = *T*_0_ + Δ*T*_t,r_, where the increase of temperature Δ*T*_t,r_ can be approximated by , which is valid for a small temperature increase Δ*T* ≪ *T*_0_ and for elongated particles with eccentricity up to 0.95.^46^ In our case, the eccentricity of the considered GNRs (sets GNR_1_ and GNR_2_) is ≈0.92.

For a metallic elongated particle dissipating a power *P*_diss_ in a surrounding medium (with thermal conductivity κ_m_), the value of the temperature excess can be estimated as^57^
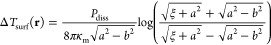
13which applies to a GNR modeled as a prolate spheroidal heat source. The term ξ = ξ(**r**, *a*, *b*) is an ellipsoidal coordinate given as a function of the Cartesian coordinates **r** = (*x*, *y*, *z*) and the parameters *a* and *b* corresponding to the major and minor semiaxis of the spheroid, respectively. In this estimation, it has been assumed that the temperature in the GNR is uniform, which is supported by the high heat conductivity of gold. The dissipation power is given by *P*_diss_ = σ_abs_(λ)*I*_trap_, with σ_abs_(λ) being its absorption cross-section at the laser wavelength. For instance, in the case of a point-like laser trap with irradiance *I*_peak_ = 6 mW/μm^2^, the surface temperature of the considered GNR_1_ is Δ*T*_surf_(ξ = 0) ≈ 122 K. Note that eq 13 is often used to estimate the temperatures of a metallic nanorod and its surrounding medium,^50^ as in our case.

We have considered this temperature-dependent motion model to predict both the translational and rotational dynamics of the GNR trapped in an off-resonant laser trap. Specifically, it requires the numerical simulation of the GNR motion described by the following Langevin equations of motion^58−60^:
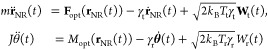
14where **r**_NR_(*t*) = (*x*(*t*), *y*(*t*)) is the position at time *t* of the GNR of mass *m* with *J* being its moment of inertia, while **ṙ**_NR_(*t*) = d**r**_NR_/d*t* is the speed vector **v**(*t*) of the GNR whose rotation angle is θ(*t*). Note that **F**_opt_ and *M*_opt_ stand for the optical force and torque exerted over the GNR (see eqs 1 and 2). The optical force term for the case of a focused point-like laser trap can be described by **F**_opt_(**r**_NR_(*t*)) = κ**r**_NR_(*t*), where κ = κ_*x*_ = κ_*y*_ is the trap stiffness along the two transverse directions (*xy* plane). This optical force arises from the intensity gradient of the focused laser beam.^17^ The terms  and  are the fluctuating thermal stochastic translational force and torque, respectively, due to the random collisions with the molecules of the surrounding medium.^17^ Note that the noise terms **W**(*t*) and *W*_r_(*t*) are assumed to be Gaussian white noise with a zero mean. Here, γ_t_ = γ_t_(*T*_t_) and γ_r_ = γ_r_(*T*_r_) are the translational and rotational drag friction coefficients, respectively. We have used analytical expressions of these drag friction coefficients for a cylinder (as our GNR) as reported in ref (39). Note that the drag friction coefficients are given as a function of the dynamic viscosity η(*T*) of the surrounding medium, which is temperature-dependent.^45^

The friction coefficients γ_t_ and γ_r_ are large enough to neglect the acceleration term; thus, the particle motion is overdamped: no average acceleration takes place, **r̈**_NR_(*t*) = 0 and θ̈(*t*) = 0. This noninertial approximation allows numerically solving eq 14 as the discrete-time sequence **r**_NR,*i*_ = (*x*_*i*_, *y*_*i*_) and θ_*i*_ at time *t*_*i*_ = *i*Δ*t* as follows:
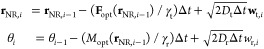
15where Δ*t* is larger than the friction relaxation times, while *D*_t_ = *k*_B_*T*_t_/γ_t_ and *D*_r_ = *k*_B_*T*_r_/γ_r_ are the diffusion coefficients for translational and rotational motions. The terms **w**_t_ = ({*w*_*x*,_}, {*w*_*y*_}) and {*w*_r_} are modeled as a Gaussian set of random numbers with zero means and unit variances. Note that the integration time step Δ*t* is smaller than the time scale on which the restoring optical force acts: Δ*t* < γ_t_/κ.^58^ In our simulations, a time step of Δ*t* = 2 μs was applied. From eq 15, it can be concluded that the expected averaged spinning frequency is *f*_avg_ = *M*_opt_/2πγ_r_ because the time average of the Brownian torque is zero. The discrete-time sequence **r**_NR,*i*_ = (*x*_*i*_, *y*_*i*_)_NR_ and θ_*i*_ are used to compute the time-series intensity fluctuations given by eq 9, used in the main text to obtain the predicted PSD and ACF.

## Supporting Information Available

Video S1: recorded video of the experiment for a GNR transported in the ring trap with linear polarization (2D trapping case); Video S2: recorded video of the experiment for a GNR transported in the ring trap with circular polarization (2D trapping case); Video S3: recorded video of the experiment for a GNR transported in the square trap with linear polarization (2D trapping case); Video S4: recorded video of the experiment for a GNR transported in the square trap with circular polarization (2D trapping case); and Video S5: recorded video of the experiment for a GNR transported in the ring trap with circular polarization (3D trapping case) (ZIP)
Synthesis of GNRs; for the GNR_1_ set: plot of the calculated longitudinal component of the GNR’s electric polarizability and of its extinction cross-section as a function of the light wavelength, plot of the corresponding normalized optical torque (for a circularly polarized laser), histograms for the measured lengths and diameters of the GNR_1_ set, and additional plots of the measured PSD and ACF; for the GNR_2_ set: plot of the calculated longitudinal component of the GNR’s electric polarizability and of its extinction cross-section as a function of the light wavelength, plot of the corresponding normalized optical torque (for a circularly polarized laser), histograms for the measured lengths and diameters of the GNR_2_ set, and additional plots of the measured PSD and ACF (PDF)
