# Supplementary material for: All-Optical Trapping and Programmable Transport of Gold Nanorods with Simultaneous Orientation and Spinning Control
Source: ACS Nano. 2024 Sep 25;18(40):27738–51. doi: 10.1021/acsnano.4c10264 (PMC11468885; doi:10.1021/acsnano.4c10264)
Supplement: Supplementary file 2 — nn4c10264_si_002.pdf [file nn4c10264_si_002.pdf]

Supplementary Information: All-optical trapping and programmable transport of gold nanorods with simultaneous orientation and spinning control

José A. Rodrigo,*,† Tatiana Alieva,† Vanesa Manzaneda-González,‡ and Andrés Guerrero-Martínez‡

†*Universidad Complutense de Madrid, Facultad de Ciencias Físicas, Ciudad Universitaria s/n, Madrid 28040, Spain*

‡*Departamento de Química Física, Universidad Complutense de Madrid, Avenida Complutense s/n, Madrid 28040, Spain*

E-mail: jarmar@fis.ucm.es

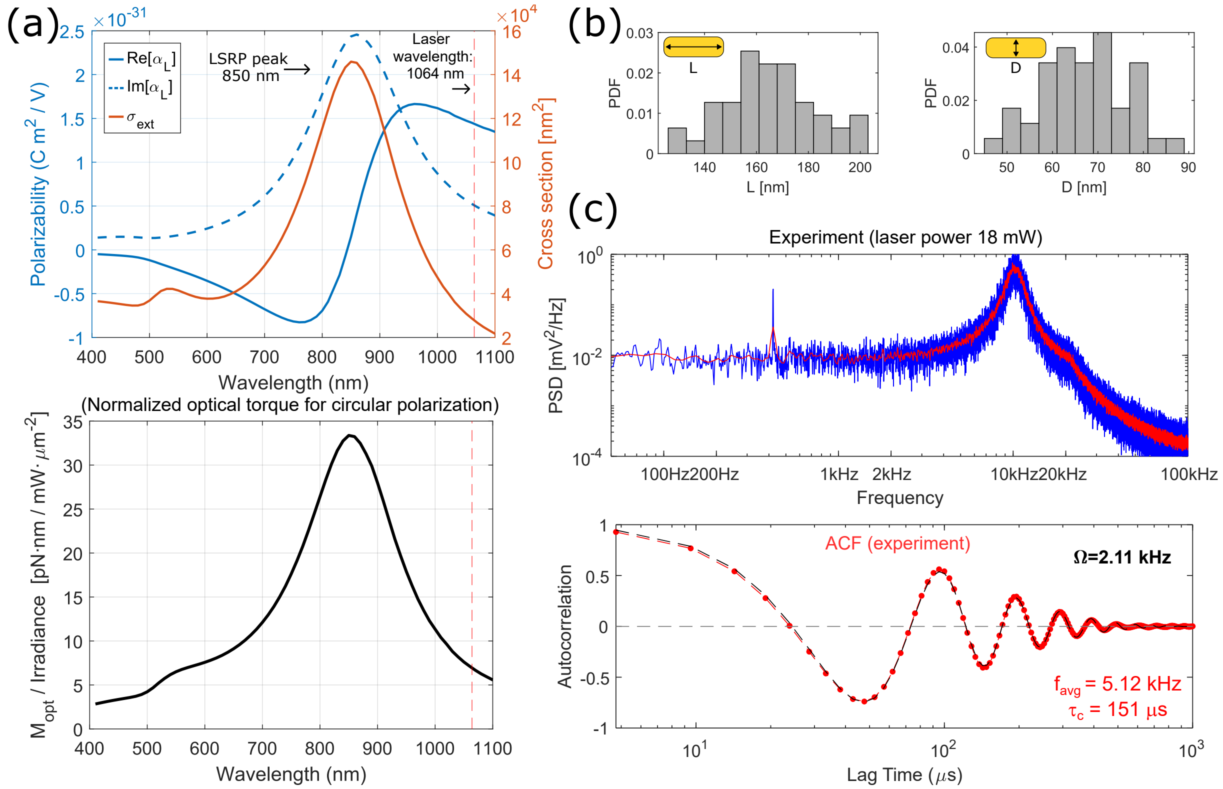

Figure 1: (a) The real and imaginary parts of the calculated longitudinal component of the GNR's electric polarizability, $\alpha_{\rm L} = \alpha'_{||}(\lambda) + {\rm i}\alpha''_{||}(\lambda)$, is displayed for the case of the representative nanorod of the GNR_1 set (size $65 \times 162\,\text{nm}^2$). Its extinction cross-section $\sigma_{\rm ext}(\lambda)$ (total, including longitudinal and traverse components) is also shown. The corresponding normalized optical torque (for a circularly polarized laser) is displayed at the second row. Note that the laser wavelength is far from the LSPR resonance of the GNR, but it is close enough to induce significant optical torque ($M_{\rm n,CP}(\lambda_{\rm laser}) = 7.1\,\text{pN}\,\text{nm}/\text{mW}\,\mu\text{m}^{-2}$). (b) Histograms for the measured lengths and diameters of the GNR_1 set. (c) Power spectral density (PSD, blue color) and auto-correlation function (ACF) of the measured intensity fluctuations. The experimental ACF (red scatter plot) fits well to the expected one (dashed black line). The ACF behavior evidences the stable rotatory motion dynamics ($f_{\rm avg} = 5.12$ kHz with $\Omega = 2.11$ kHz and $\tau_c = 151\,\mu$s) of a GNR trapped in 2D (the same GNR as in the Figure 1a of the main text), under a laser power of 18 mW. The averaged PSD displayed in red color evidences the persistence of the measured signal. Let us recall that the small peak observed at frequency $f_{\rm SLM} = 422$ Hz corresponds to residual intensity fluctuations caused by the SLM device operation.

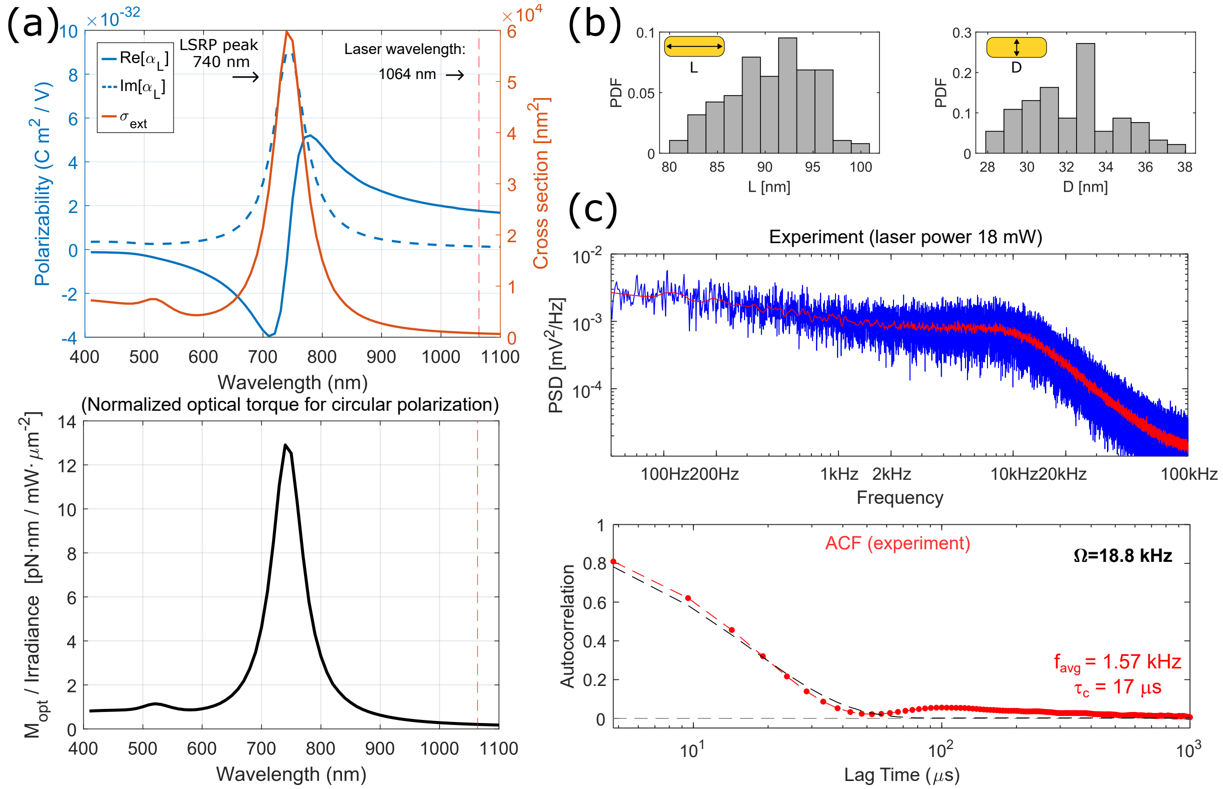

Figure 2: (a) The real and imaginary parts of the calculated longitudinal component of the GNR's electric polarizability, $\alpha_{\mathrm{L}} = \alpha'_{\|}(\lambda) + \mathrm{i}\alpha''_{\|}(\lambda)$, is displayed for the case of the representative nanorod of the GNR_2 set (size $33 \times 90\,\mathrm{nm}^2$). Its extinction cross-section $\sigma_{\mathrm{ext}}(\lambda)$ (total, including longitudinal and traverse components) is also shown. The corresponding normalized optical torque (for a circularly polarized laser) is displayed at the second row. Note that the laser wavelength is now significantly far form the LSPR resonance of the GNR, and the optical torque is weaker ($M_{\mathrm{n,CP}}(\lambda_{\mathrm{laser}}) = 0.2\,\mathrm{pN\,nm/mW}\,\mu\mathrm{m}^{-2}$). (b) Histograms for the measured lengths and diameters of the GNR_2 set. (c) Power spectral density (PSD, blue color) and auto-correlation function (ACF) of the measured intensity fluctuations. It evidences the unstable rotatory motion dynamics of a GNR trapped in 2D, under the same laser power of 18 mW considered in Figure S1. The averaged PSD displayed in red color evidences the persistence of the measured signal. The spinning of the GNR is hindered by the rotational Brownian fluctuations, which explains the observed large peak brocading ($\Omega = 18.8$ kHz) in the PSD and the short decay correlation time $\tau_c = 17\,\mu$s (see ACF).

Synthesis of Gold Nanorods (GNRs)

All chemicals used were procured from Sigma-Aldrich, including cetyltrimethylammonium bromide (CTAB, ≥99%), cetyltrimethylammonium chloride (CTAC, 25% w/w aqueous solution), n-decanol (98%), gold(III) chloride trihydrate ($HAuCl_4 \cdot 3H_2O$, ≥99.9%), silver(I) nitrate ($AgNO_3$, ≥99.0%), L-ascorbic acid (≥99%), and sodium tetrahydroborate ($NaBH_4$, 99%). Deionized water, with a resistivity of 18.2 MΩ·cm at 25 °C, was used for nanoparticle synthesis.

Preparation of 1–2 nm Au Seeds

Gold seeds were synthesized using a modified seeded growth method, as described in reference 40 of the main text. To prepare the seeds, 200 µL of a 0.05 M $HAuCl_4$ solution and 100 µL of a 0.1 M ascorbic acid solution were added with gentle stirring to 20 mL of a 50 mM CTAB and 13.5 mM n-decanol solution. The temperature was maintained at 25 °C. After 2 minutes, 800 µL of a freshly prepared 0.02 M $NaBH_4$ solution was added to the colorless solution under vigorous stirring, resulting in a brownish-yellow solution. The seed solution was allowed to age for 1 hour at 25 °C before use.

Preparation of Small Anisotropic Seeds

For the synthesis of small anisotropic seeds, 300 mL of a 50 mM CTAB and 11 mM n-decanol solution was placed in a 500 mL Erlenmeyer flask. Sequential additions included 3000 µL of 0.05 M $HAuCl_4$, 2400 µL of 0.01 M $AgNO_3$, 21 mL of 1 M HCl, and 3900 µL of 0.1 M ascorbic acid. The solution was maintained at 25 °C throughout the process. Subsequently, 18 mL of the seed solution was added under stirring. The mixture was left undisturbed at 25 °C for at least 4 hours, during which time the solution transitioned from colorless to a dark brownish-gray hue. The resulting small anisotropic seeds, with a longitudinal LSPR at 726 nm, were then centrifuged at 14,000–15,000 rpm for 60 minutes. The precipitate was

redispersed in 100 mL of a 10 mM CTAB solution and centrifuged again under the same conditions. The final gold concentration was adjusted to 4.65 mM (Abs_{400nm}: 1, optical path: 0.1 cm).

Preparation of Gold Nanorods (GNRs)

In a typical synthesis, 2500 µL of a 0.01 M $AgNO_3$ solution, 1000 µL of a 0.05 M $HAuCl_4$ solution, 9 (for smaller GNRs) -10 (for larger GNRs) mL of a 1 M HCl solution, and 800 µL of a 0.1 M ascorbic acid solution were added under stirring to 100 mL of a 50 mM CTAB and 11 mM n-decanol solution maintained at 25 °C. Following this, 0.25 (for larger GNRs) - 0.5 (for smaller GNRs) mL of the small anisotropic seed suspension was introduced under stirring. The mixture was left undisturbed for 6 hours to allow growth. After synthesis, the GNRs were isolated by centrifugation at 7000 rpm for 30 minutes to remove excess surfactant, resulting in a sediment. The sediment was then redispersed in 10 mL of a 25 mM CTAC solution to form the GNR stock solution. This cleaning procedure was repeated twice to ensure removal of CTAB traces. Finally, the GNRs were redispersed in 2.5 mL of a 25 mM CTAC solution.
